# A Systematic Review of the Gastrointestinal Microbiome: A Game Changer in Colorectal Cancer

**DOI:** 10.7759/cureus.28545

**Published:** 2022-08-29

**Authors:** Aziza K Eastmond, Chaitra Shetty, Syed Muhammad Hannan Ali Rizvi, Joudi Sharaf, Kerry-Ann D Williams, Maha Tariq, Maitri V Acharekar, Sara Elena Guerrero Saldivia, Sumedha Unnikrishnan, Yeny Y Chavarria, Adebisi O Akindele, Ana P Jalkh, Prachi Balani

**Affiliations:** 1 Research, California Institute of Behavioral Neurosciences & Psychology, Fairfield, USA; 2 Internal Medicine, California Institute of Behavioral Neurosciences & Psychology, Fairfield, USA; 3 Internal Medicine, Saint Vincent Hospital, Worcester, USA

**Keywords:** microbiome, gastrointestinal, colorectal neoplasm, colorectal cancer, gut microflora, gut microbiome, gastrointestinal microbiome

## Abstract

Colorectal cancer (CRC) is a malignant condition of the colon and rectum. Generally, malignancies constitute a significant health threat to humans, and the result can be devastating. CRC is no exception. The gastrointestinal (GI) microbiome has long been suspected of impacting CRC. This review seeks to explore whether there is a connection between the two or not. For screening purposes, relevant articles were culled from various databases using key terms and phrases. Following a thorough search, the inclusion and exclusion criteria were applied, and a quality assessment was conducted. The articles retained were comprehensively studied, and revealed imbalances of the GI microbiome do indeed exhibit an association with CRC.

## Introduction and background

Cancers impose a tremendous burden on the healthcare system, colorectal cancer (CRC) being the third most common worldwide [1]. The evolution of CRC consists of interconnected parts, including inflammation, gene mutations, non-modifiable risk factors such as a known family history of CRC and advanced age, and modifiable risk factors such as obesity, excessive alcohol and tobacco use, and diets high in processed and red meat [2]. Interestingly, the USA statistics infer that different populations, including multiple ethnicities with different lifestyles, still suffer significantly from high morbidity and mortality [1].

Despite an abundance of knowledge on the causation of CRC, a great deal remains unknown. Particularly its correlation with the gastrointestinal (GI) microbiome. The GI microbiome is composed of multifarious inhabitants of microorganisms in the GI system, including bacteria, viruses, fungi, protozoans, and archaea [3]. Healthy individuals possess a colonic microbiome, predominantly composed of Gram-negative Bacteroidetes and Gram-positive Firmicutes, with a lower frequency of Actinobacteria and Verrucomicrobia [4]. The GI microbiome inaugurates a defensive mechanism against infection and is involved in specific preservative and metabolic functions within the intestinal epithelium and GI homeostasis maintenance [5].

Some studies suggest that the GI microbiome is predictive of CRC [6], and others have discovered that it possibly has a role in causing CRC [7,8]. Most of these studies display this correlation in animal studies, with few providing limited application to humans [7]. Further exploration into human investigation and analysis is necessary to confirm the connection between CRC and the immune response [2].

The GI tract is an extensive organ system that starts from the mouth and ends at the anus. Naturally, different parts of the tract will have differing microbiome compositions. Therefore, there is a possibility that each piece may have an alternative contribution to the development of CRC. The emergence of CRC at specified colorectal sites occurs via different molecular pathways. Although different bacteria may be engaged in each process and are linked to different precursors and locations, there is limited evidence proving that the GI microbiome is linked to colorectal polyp occurrence by histology or site [7].

Probiotics include Lactobacilli, which inhibits the growth of colonic carcinoma cells [9]. Consumption of probiotics may be our reedmace for maintaining a balanced GI microbiome and, therefore, optimistically, may assist with CRC prevention. A more impactful emphasis needs to be placed on prevention rather than its cure and treatment, especially for modifiable risk factors that are partly in the control of the population at risk. This study is a systematic review that will determine if the GI microbiome culminates in the causation and, by extension, prevention of CRC in the human species.

Methods

We used the Preferred Reporting Items for Systematic Reviews and Meta-Analyses (PRISMA) guidelines [10]. The Cochrane Risk Assessment bias tool, the Scale for the Assessment of Narrative Review Articles (SANRA) checklist, the Joanna Briggs Institute (JBI) check tool, and the Newcastle-Ottawa tool were utilized for quality assessment checks.

**Figure 1:**
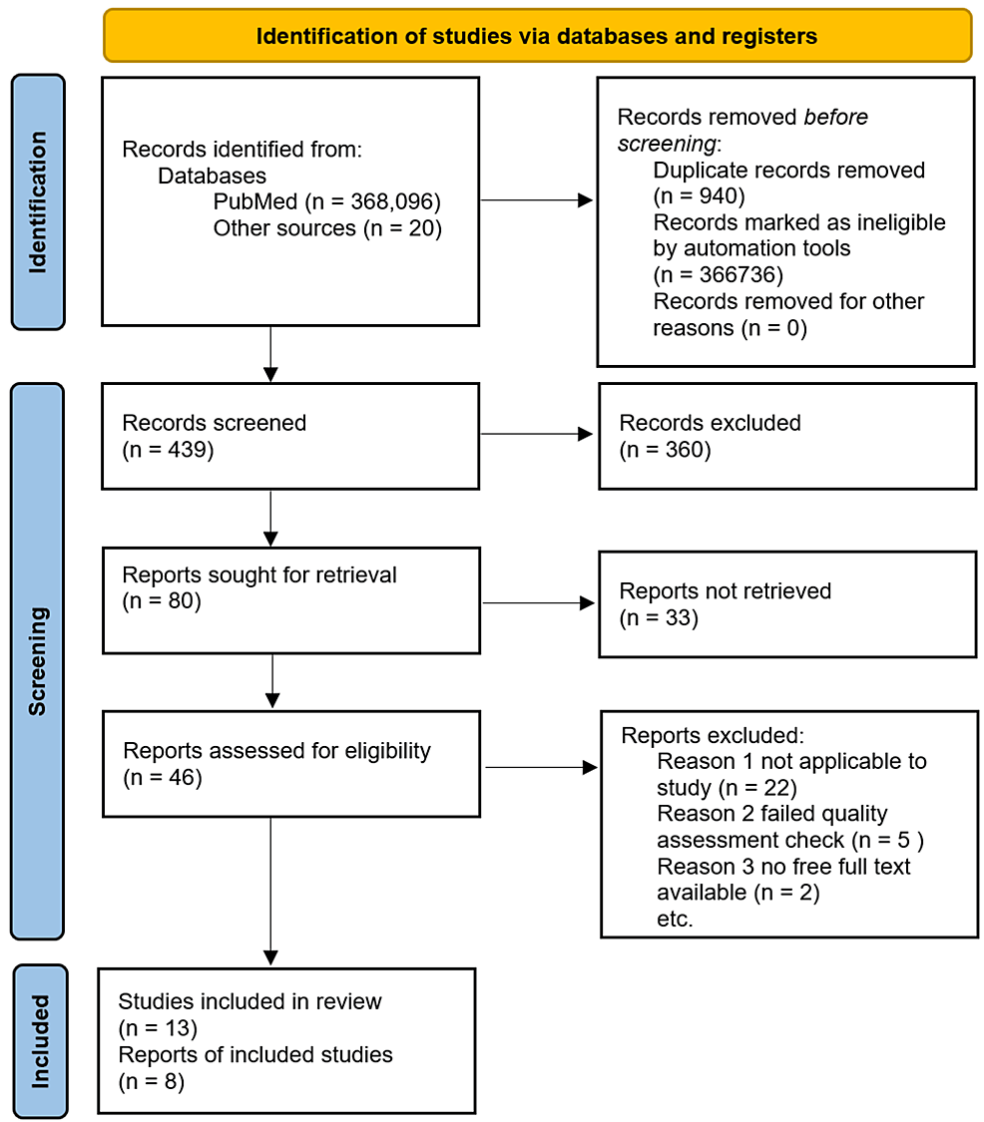
The PRISMA flowchart PRISMA: Preferred Reporting Items for Systematic Reviews and Meta-Analyses.

The PRISMA flow chart can be seen in Figure 1. PubMed, PubMed Central, and MEDLINE were searched for relevant literature about concepts one and two separate and, subsequently, concepts one and two combined. See Table 1 for how the concepts were used to collect articles.

**Table 1:** The search strategy used to obtain articles for review from different databases

Search strategy	Concept 1	Concept 2	Concepts 1 and 2
	Colorectal cancer	Gastrointestinal microbiome	Colorectal cancer and gastrointestinal microbiome
Database	PubMed and MEDLINE	PubMed and MEDLINE	PubMed and MEDLINE
No. of articles	59,346	34,448	274,302

Of note, an additional 20 articles were obtained from the reference section of several articles upon quality assessment check from the same database (PubMed).

After that, keywords, including ("Colorectal Neoplasms/prevention and control"[Majr]) AND "Gastrointestinal Microbiome/drug effects"[Majr] OR Gut Microbiome OR Gut Microflora OR Gut Microbiota OR Gastrointestinal Flora OR Gut Flora OR Gastrointestinal Microbiota OR Gastrointestinal Microbial Community OR Gastrointestinal Microflora OR Gastric Microbiome OR Intestinal Microbiome OR Intestinal Microbiota OR Intestinal Microflora OR Intestinal Flora OR Enteric Bacteria, were used in the search.

The following inclusion and exclusion criteria were implemented for the search: free full text, meta-analysis, clinical trial/observational study, randomized controlled trial, systematic review, the timeline between the years 2018 and 2022, human and animal species, the English language, MEDLINE, and adult (19+ years). Duplicates were then removed.

Results

A total of 368,096 publications were retrieved from PubMed, PubMed Central, and MEDLINE searches. After implementing the inclusion and exclusion criteria, 1360 articles were left, and 940 duplicates were removed. Subsequently, 420 articles were screened by title. An additional 20 articles were obtained from reference sections of other studies, bringing the number to 440 studies for review by abstract. Of these, 80 articles were screened by review of free full text and quality assessment tool application and yielded 46 eligible articles. A total of 13 studies were included in our systematic review. The articles used for review incorporate one statistical journal, one case-control, one meta-analysis, two observational studies, and eight review studies. The review has sample sizes from eight of these studies with a total number of 3012 patients. The PRISMA flowchart can be seen in Figure 1.

## Review

Discussion

The disordered physiology of the GI tract should be highlighted and examined to fully understand the carcinogenesis of the colon and rectum.

Pathophysiology of CRC

**Figure 2:**
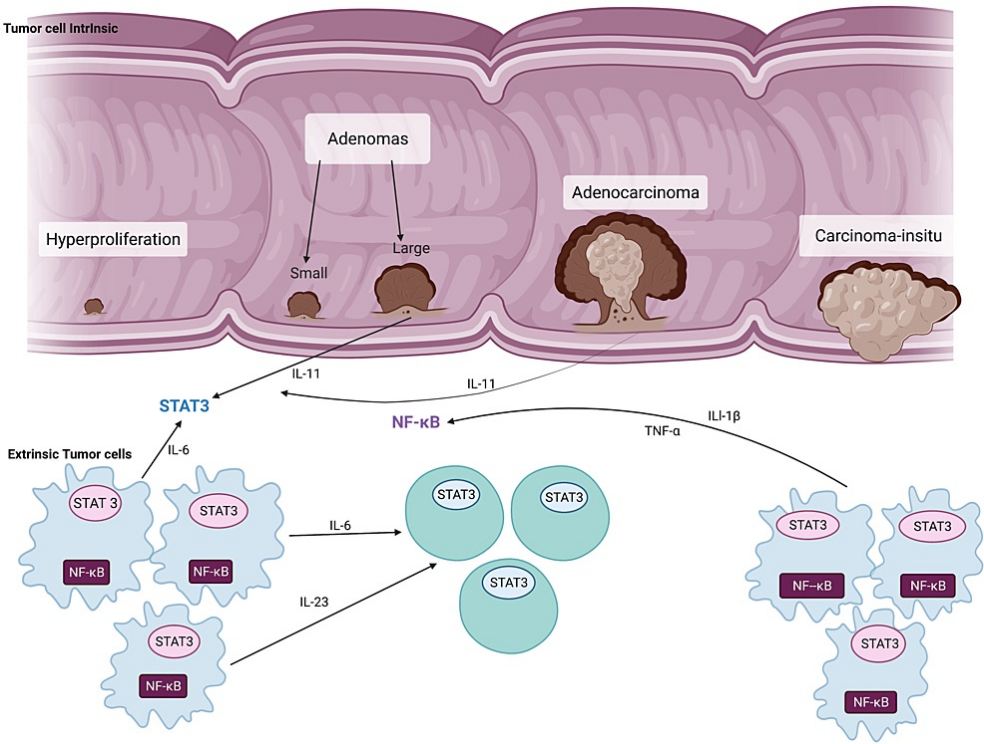
The pathophysiology of colorectal cancer The blue cells represent myeloid cells and the green cells represent lymphoid cells. Adapted from the article entitled "Targeting IL-11 signaling in colon cancer." We used BioRender.com to create this diagram [11,12]. IL-11: interleukin 11; IL1-β: interleukin 1 beta; IL-6: interleukin 6; IL-23: interleukin 23; TNF-α: tumor necrosis factor-alpha; STAT 3: signal transducer and activator of transcription 3; NF-κΒ: nuclear factor kappa light chain enhancer of activated B cells.

The genesis of CRC commences with increased proliferation of the GI epithelium resulting in a disorganized structure forming adenomas that become incorporated into the submucosa and transform into carcinoma. This uncontrolled proliferation is influenced by specific pathways that are pro-inflammatory. Mainly, cytokines such as tumor necrosis factor-alpha (TNF-α), interleukin 6 (IL-6), interleukin 8 (IL-8), and interleukin 1 beta (IL-1β) are involved in this adenocarcinoma sequence of CRC by conduction of nuclear factor-kappa B (NF-κB) and signal transducer and activator of transcription 3 (STAT3) [2,3] signal cascading pathways (see Figure 2).

Like any other organ in the human body, the GI system has an environment of microorganisms. Its’ composition, or in other words, microbiome, is pertinent to the special functions it conducts.

The Normal GI Microbiome

The normal GI microbiome consists of Firmicutes and Bacteroidetes, anaerobic phyla, Actinobacteria, and Verrucomicrobia to a lesser extent. It protects healthy persons through its metabolic and immunologic mechanisms [3]. The research proposes that the GI microbiome can act as an indicator of CRC [6] and possibly causes it [7,8]; for example, the fermentation of carbohydrates creates short-chain fatty acids (SCFAs) that are utilized as energy by the host; however, this fermentation can produce toxins such as phenols, ammonia, and sulfides, which may be carcinogenic [4]. Table 2 can be seen here, which describes the GI microbiome as an essential denominator in CRC disease development.

**Table 2:** Modalities of GI microbiome disruption, toxic formation, colorectal adenoma transformation, and CRC development GI: gastrointestinal; CRC: colorectal cancer; FOBT: fecal occult blood testing; SCFAs: short-chain fatty acids.

Study	Author	Year	Type of study	Patients	Purpose of the study	Results	Conclusion
1	Alhinai et al. [4]	2019	Review		Determine the role of the GI microbiome in contributing to colorectal carcinogenesis.	Enterotoxic strains of *Bacteroides fragilis*, certain strains of *Escherichia coli*, *Fusobacterium nucleatum*, *Enterococcus faecalis*, and *Streptococcus gallolyticus* are associated with CRC.	GI microbes are helpful because they have different roles in various carcinogenic pathways, from inflammation to immunosuppression.
2	Wirbel et al. [6]	2019	Meta-analysis	768	Delineate links between numerous studies with fewer biological or technical confounders compared to the previous meta-analysis.	The degree to which the GI microbiome causes disease, specifically colorectal carcinogenesis, was quantified. Examples include *Fusobacterium nucleatum*, the *Bacteroides fragilis* enterotoxin (bft gene), and colibactin produced by some *Escherichia coli* strains (polyketide synthases genomic island).	The study demonstrates the pathogenic features within this study and other datasets and offers promising potential for determining the impact of the GI microbiome on CRC development.
3	Yang et al. [8]	2019	Systematic review	100	To identify if the GI microbiome and total collective metabolites of human CRC fecal samples can depict an association between each other.	Polyamines are potential biomarkers for CRC, just as FOBT (a modality already used for CRC screening).	The fecal microbiome has an uneven and less microbial variability in CRC.
4	Lucas et al. [3]	2017	Review		Create an outline of the links between the GI microbiome and CRC, explicitly focusing on the pro-carcinogenic properties of bacteria. Additionally, delineate management options based on manipulation of the GI microbiome.	Treatment modalities such as SCFAs, prebiotic and probiotic consumption, and activation of anti-cancer immunity or suppression of signaling pathways involved in carcinogenesis can be adopted.	Attenuation of the GI microbiome depicted can provide homeostasis between the GI tract, the immune system, and the microbiome.
5	Peters et al. [7]	2016	Systematic review	540	Study the relationship of the GI microbiome to different colorectal polyp types.	A comparison was made between the GI microbiome composition of individuals with polyps and those without, revealing a collaboration in the early stages of colorectal carcinogenesis through the development of conventional adenomas.	There is a potential for CRC prevention by focusing on early microbial factors contributing to colorectal carcinogenesis.

The alterations of the GI microbiome are postulated to have an outcome of CRC. Some studies have explored this idea and have provided statistics.

Dysbacteriosis and Its Correlation With CRC

CRC development is influenced by multiple factors, namely, genetic, environmental, and lifestyle. Disruption of the GI microbiome is another critical factor. Various studies have proven that specific microbes are correlated with an increased incidence of CRC.

Zhang et al. discovered that 14 of 24 species used in their study were increased in CRC patients, including *Parvimonas micra*, *Solobacterium moorei*, and *Clostridium symbiosum* [2]. Yu et al. found similar findings specifically with these three organisms. Furthermore, *Parvimonas micra* had a 72.2% occurrence in patients with CRC compared to a 50% occurrence in controls [13]. In contrast, *Solobacterium moorei* demonstrated a 66.7% occurrence in patients with CRC compared to 27.7% occurrence in controls as per Osman et al. [14]. According to Lucas et al. [3], *Bacteroides fragilis* has a 38% occurrence in CRC and 12% in controls. Osman et al. showed a higher percentage of occurrence of *B. fragilis* in CRC at 77.8% [14]. Overall, changes within the GI microbiome, particularly a high prevalence of specific organisms, can be detrimental, leading to colorectal tumorigenesis.

Moreover, data show the GI microbiome has a role in CRC carcinogenesis at different stages. Coker et al. discovered that the altered GI microbiome demonstrates CRC (p = 0.0006) with greater statistical significance than control groups (p = 0.001) [15]. The fungi, *Ascomycota* and *Basidiomycota* being the most abundant among others, portrayed different clusters for control, early-stage (tumor, node, and metastasis (TNM) stage I and II) CRC, and late-stage (TNM stage III and IV) CRC, depicting a CRC-stage-specific disruption in microbiome homeostasis.

Zhang et al. determined that there is disruption of the symbiosis of the oral microbiome in the colorectal adenoma (CRA) and CRC, as evidenced by an imbalance in phyla composition and diversity as well as function [16]. Additionally, the probability of disease (POD) index for CRA and CRC was achieved with an area under the curve (AUC) value of 95.94% (95% CI: 90.83%-100%). See Table 3 below, which determines the possible culprits responsible for GI microbiome changes and describes how they expedite CRC progression.

**Table 3:** Microbes highlighted and likely responsible for CRC development GI: gastrointestinal; CRC: colorectal cancer; CRA: colorectal adenoma; SCFA: short-chain fatty acid.

Study	Author	Year	Type of study	Patients	Purpose of the study	Results	Conclusion
1	Osman et al. [14]	2021	Observational study	36	To identify the GI microbiome of Malaysian CRC patients.	All bacteria in the study, *Parvimonas micra*, *Peptostreptococcus stomatis*, *Fusobacterium nucleatum*, and *Akkermansia muciniphila*, show statistical significance (p ≤ 0.001) in the CRC patients, all of which had a surplus of 66% of these bacteria. In comparison, non-CRC controls had only less than 30%.	Malaysian CRC patients have a high incidence of bacteria.
2	Zhang et al. [16]	2020	Observational study	253	To determine if the oral microbiome has an association with CRC and if it can be used as a biomarker for CRC.	Higher variegation of the oral microbiome in CRA has shown to be an increased risk for GI tumorigenesis.	Biomarkers of the oral microbiome may be predictive of the development of CRA and CRC.
3	Coker et al. [15]	2019	Systematic review	585	Denote the GI microbiome in CRC.	Ascomycota and Basidiomycota demonstrate an association with CRC.	Dysbiosis of fungi in the GI tract is relevant to CRC development.
4	Zhang et al. [2]	2018	Case-control	410	The correlation of plasma inflammatory factors and CRC-associated bacteria was explored, and their modifications individually in the evolution of the adenoma-carcinoma sequence.	Numerous CRC-associated bacteria were abundant in CRC patients, and inflammatory factors play an essential role in the relationship between bacterial pathogens and CRC.	The GI microbiome and inflammation can potentially promote the development of CRC.
5	Lucas et al. [3]	2017	Review		Create an outline of the links between the GI microbiome and CRC, explicitly focusing on the pro-carcinogenic properties of bacteria. Additionally, delineate management options based on manipulation of the GI microbiome.	Treatment modalities such as SCFA, prebiotic and probiotic consumption, and activation of anti-cancer immunity or suppression of signaling pathways involved in carcinogenesis can be adopted.	Attenuation of the GI microbiome depicted can provide homeostasis between the GI tract, the immune system, and the microbiome.
5	Yu et al. [13]	2017	Systematic review	284	To determine if the fecal microbiome has the potential for diagnosing CRC.	*Parvimonas micra*, *Solobacterium moorei*, and *Fusobacterium nucleatum* were discovered in CRC patient microbiomes across populations suggesting that they may be used as biomarkers and have better diagnostic potential than control-enriched biomarkers.	Phyla of the fecal microbiome are involved in the development and progression of CRC.

Limitations

This systematic review divulges the correlation between the GI microbiome and CRC by expressing the quantity of alteration in the GI microbiome as seen by the high occurrence of various species but does not explicitly state how the change can be avoided to halt the progression of CRC. Additionally, the studies used were all reviews, therefore, pertinent research articles, some basic science and animal studies that look at the basic mechanism and link between the gut microbiome and cancer, as well as other clinical studies were omitted. Furthermore, the selection of only free articles may have led to the exclusion of relevant papers.

## Conclusions

This systematic review targeted the GI microbiome and sought to determine if any changes within it cause CRC. The ways CRC evolves include inflammation and immunosuppression, as seen in the research. The review demonstrates that changes in the microbiome at different parts of the GI tract, including the oral cavity, are responsible for the progression of CRA and CRC, acting as predictors for morbidity. Additionally, fecal matter diversity is limited in CRC.

However, the study did not adequately delineate how adenoma and carcinoma formation can be circumvented. Further research needs to be implemented in GI microbiome homeostasis into CRC prevention and treatment. If proven to provide adequate management of CRC, it could lead to better outcomes, especially since it is less invasive, cost-effective, and easy for patients and healthcare providers.
